# QUANTIFICATIONS OF BIOLOGICAL AGING PREDICT DISABILITY AND MORTALITY IN OLDER ADULTS IN THE DUKE EPESE

**DOI:** 10.1093/geroni/igz038.343

**Published:** 2019-11-08

**Authors:** Daniel C Parker, Virginia B Kraus, Daniel Belsky

**Affiliations:** 1 Duke University School of Medicine, Durham, North Carolina, United States; 2 Duke University, Durham, North Carolina, United States; 3 Columbia University Mailman School of Public Health, New York, New York, United States

## Abstract

Methods to quantify biological aging have been proposed to measure age-related decline in system integrity for population surveillance and evaluation of geroprotective therapies. However, quantifications of biological aging have been little-studied in geriatric populations. We conducted analysis of three clinical-biomarker-algorithm methods to quantify biological aging, the Klemera-Doubal Method (KDM) Biological Age, homeostatic dysregulation (HD), and Levine Method (LM) Biological Age in a cohort of N=1,374 older adults aged 71-102 years (35% male, 52% African American), the Duke-EPESE. We parameterized algorithms from analysis of US NHANES data (N=36,207). We conducted criterion validity analyses using measures of disability and mortality as end-points. We analyzed counts of ADLs and iADLs using negative binomial regression. We analyzed time-to-death using Cox regression. Models were adjusted for age, sex, and race/ethnicity. We evaluated algorithms derived from analysis of different biomarker groupings. We also compared algorithms derived from analysis of mixed age and race/ethnicity samples to algorithms derived from older-age (65+) and individual race/ethnicity samples. Duke-EPESE participants with older KDM Biological Age reported dependence in more ADLs and iADLs, and were at increased risk of death (ADL IRR=1.19 [1.12, 1.27]; IADL IRR=1.18 [1.10, 1.26]; mortality HR=1.09 [1.06, 1.13]). Quantifications of biological aging derived from analysis of a mixed-age and race/ethnicity sample predicted disability and mortality in African-American and white older adults.

